# Seizure detection using wearable electrocardiogram connected to a smartphone: a phase 3 clinical validation study

**DOI:** 10.1016/j.ebiom.2025.105952

**Published:** 2025-09-29

**Authors:** Jesper Jeppesen, Jakob Christensen, Oliver Ahrenfeldt Petersen, Sarah Fenger, Sidsel Armand Larsen, Stephan Wüstenhagen, Stefan Rahr Wagner, Peter Johansen, Sándor Beniczky

**Affiliations:** a Department of Clinical Neurophysiology, Aarhus University Hospital, Aarhus, Denmark; b Department of Clinical Medicine, Aarhus University, Aarhus, Denmark; c Department of Neurology, Aarhus University Hospital, Aarhus, Denmark; d Department of Electrical and Computer Engineering, Aarhus University, Aarhus, Denmark; e Department of Clinical Neurophysiology, Danish Epilepsy Centre, Dianalund, Denmark

**Keywords:** Automated behavioural testing, Automated seizure detection, Electrocardiography, Focal seizures, Heart rate variability, Tonic-clonic seizures

## Abstract

**Background:**

Automated seizure detection is needed for patient safety and for objective seizure quantification. Wearable seizure detection devices hold great potential to improve patient care. Our objectives were to assess the accuracy of a wearable ECG-device connected to a smartphone, in detecting epileptic seizures in patients with autonomic ictal changes, and evaluate its capability to automatically determine impairment of consciousness.

**Methods:**

We conducted a phase 3, prospective, blinded, multicentre, clinical validation study of real-time seizure detection using a predefined algorithm. We recruited consecutive patients admitted to Epilepsy Monitoring Units. Eligible patients experienced seizures with autonomic ictal manifestations, defined as ictal heart rate change exceeding 50 beats per minute, inferred from the first recorded seizure. Patients wore an ECG-device connected to a smartphone. The algorithm, based on heart rate variability, used a personalised detection threshold determined from the first 24 h of recording. During daytime, seizure detection triggered automated behavioural-testing on the smartphone to confirm detection and assess consciousness.

**Findings:**

Of 101 enrolled patients, 36 experienced seizures, with 42 seizures recorded from 17 eligible patients. Overall sensitivity across all 42 seizures was 90·5% (95% CI: 77·4–97·3%), median sensitivity per patient was 100% (95% CI: 100–100%). All bilateral tonic-clonic seizures were detected, while sensitivity for other focal seizures was 82·6% (95% CI: 61·2–95·1%), median per patient: 100% (95% CI: 60–100%). Mean false alarm rate was 2·5/day (median per patient: 1·1/day, 95% CI: 0–2·8/day, zero during the night). Device deficiency time was 1·8% and signal loss was 4·5% (median per patient: 0·3% and 0·5% respectively). Use of the behavioural-testing application successfully cancelled all false alarms and accurately identified impairment of consciousness.

**Interpretation:**

The wearable ECG device connected to a smartphone accurately detected focal and generalised seizures, and assessed impairment of consciousness.

**Funding:**

10.13039/501100011958Independent Research Fund Denmark (grant number 0134-00400B).

**Research in context**

**Evidence before this study**

We searched in PubMed and EMBASE for publications from database inception to January 1, 2025, using the keywords: ((automated detection) OR (algorithm AND detection) OR (wearable AND detection)) AND (epilepsy OR seizure), not limited to English language. We identified phase 3 clinical validation studies on automated seizure detection using non-invasive wearable devices. Five studies reported detection of generalised tonic-clonic seizures, while one study examined the detection of absence seizures.

A recent clinical practice guideline from the International League Against Epilepsy and the International Federation of Clinical Neurophysiology stated that evidence is lacking on the reliability of wearable devices for detecting seizures other than generalised tonic-clonic ones. It emphasised the need for further research and development, particularly for seizures without generalised convulsions.

**Added value of this study**

In this phase 3 clinical validation study we found that a wearable ECG-device connected to a smartphone detected all bilateral tonic-clonic seizures and 82·6% of the focal seizures with autonomic ictal changes (overall sensitivity: 90·5%) with a median false alarm rate of 1·1/24 h (zero during night). The automated behavioural-testing cancelled all false alarms and identified all seizures with impaired consciousness. The average seizure frequency was 1·14 seizures per 24 h.

**Implications of all the available evidence**

The wearable ECG-device connected to a smartphone may enhance patient safety and provide accurate seizure quantification for patients with autonomic ictal changes (nearly half of those with epilepsy).

## Introduction

Automated seizure detection using wearable devices is needed for patient safety and objective quantification of seizure burden.^1^, ^2^, ^3^ According to clinical practice guidelines from the International League Against Epilepsy and the International Federation of Clinical Neurophysiology, validated wearable devices currently are recommended only for safety indications—i.e., for detecting tonic-clonic seizures (TCS), including focal-to-bilateral tonic-clonic seizures, which are associated with the highest morbidity and mortality.^1^^,^^2^^,^^4^ However, detection of the other seizure types is necessary for objective seizure quantification,^1^^,^^2^^,^^5^ as the current clinical approach relies on self-reporting by patients and caregivers, which is often inaccurate due to both underreporting and overreporting of seizure frequency.^1^^,^^2^^,^^5^^,^^6^ Consequently, therapeutic decisions and clinical drug trials are based on inaccurate data. Moreover, timely seizure alerts may help mitigate seizure-related morbidity.^1^^,^^2^^,^^7^

In previous studies, we identified the modified cardiac sympathetic index (CSI), a heart rate variability (HRV) measure, as a biomarker for seizures involving the autonomic nervous system—present in 50–80% of patients with epileptic seizures.^8^^,^^9^ HRV refers to the fluctuation in the time intervals between heartbeats. Unlike tachycardia, which can occur under physiological conditions such as physical activity and may lead to false-positive detections, the modified CSI is specific to autonomic nervous system changes that occur during seizures.^8^^,^^9^ We demonstrated that patients with ictal autonomic involvement, and thus eligible to HRV-based seizure detection, could be identified by an ictal heart rate increase of >50 beats/min. Identification of patients with ictal heart rate increase and therefore eligible for HRV-based seizure detection is feasible in a home environment using commercially available smartwatches. In phase-2 studies, we showed that our algorithm, based on HRV data from wearable ECG devices, reliably detected focal seizures in patients with autonomic changes during seizures, achieving a sensitivity of 84·6%–90·6% and a false alarm rate (FAR) of 1 per 24 h.^10^^,^^11^ However, these studies conducted seizure detection analysis retrospectively, using ECG recordings collected prospectively from patients.

The objective of this phase-3,^12^ prospective, blinded, multicentre clinical validation study was to evaluate the accuracy of automated seizure detection using a wearable ECG device connected to a smartphone in patients with ictal autonomic changes. A predefined (“fixed and frozen”) algorithm, based on CSI, performed real-time automated seizure detection, triggering behavioural-testing via a smartphone application to assess patient responsiveness, recall (awareness) and cancel false alarms. The diagnostic reference standard (gold standard) was expert evaluation of video-EEG recordings. Analysis of the index test (automated seizure detection using CSI) and investigator managing the seizure detection device were blinded to the reference standard.

## Methods

### Patients

We prospectively recruited consecutive patients aged 4 years and older, with epilepsy, undergoing long-term video-EEG monitoring for diagnostic purposes or presurgical evaluation at the epilepsy monitoring units (EMU) of the Danish Epilepsy Centre, Dianalund, and Aarhus University Hospital, Denmark, from February 2022 to September 2024. To ensure a realistic assessment, patients were allowed maximum flexibility in their daily routines, including meal times, bedtimes, and mobility during the study. Patients had a large degree of mobility in the EMU: wireless amplifiers and the EMU designed specifically for this purpose allowed patients to move freely within their room and within the EMU which at the Danish Epilepsy Centre included a living room and a dining room, a space for physical exercise (treadmill, indoors bike), playground for children, and a patio with rubber floor.^13^ Inclusion criteria: epilepsy diagnosis, and referral for video-EEG monitoring. Exclusion criteria: implanted electronic devices (e.g., pacemakers or vagus nerve stimulators, due to potential ECG interference), and pregnancy.

### Study design

The study strictly followed the guidelines of a phase-3 clinical study for testing and validation of seizure detection devices as it has been outlined in “Standards for testing and clinical validation of seizure detection devices”.^1^^,^^12^ This entails: 1) recruiting a minimum number of 20 patients with seizure, 2) recording at least 30 seizures, 3) using a dedicated device, 4) continues measurement, 5) multicentre study, 6) a predefined algorithm and cutoff values, 7) real-time seizure detection, 8) reference standard being video-EEG recordings. Sample size estimation was based on fulfilling the criteria's of a phase-3 study.^12^

### Ethics

Written informed consent was obtained from all participants or, for patients under 18 years of age assent was sought along with parental consent. The study was approved by the Danish National Medical Research Ethics Committees (ID nr 2119788) prior to the recruiting of the patients. The study was registered on the International Standard Randomised Controlled Trial Number Registry with the following reference number: ISRCTN85640482 (https://doi.org/10.1186/ISRCTN85640482). We report the study according to the Standards for Reporting Diagnostic Accuracy Studies.^14^^,^^15^

### Tasks

To establish each patient's physiological HRV, participants were asked on the first day of monitoring to complete three tasks: a 3-min all-out exercise bike test, a 10-min Paced Auditory Serial Addition Test (PASAT)^16^ to induce cognitive stress, and a horror movie viewing session (adult patients only). Participation in these activities was optional to maximise patient recruitment.

### The wearable device and the smartphone application

**Fig. 1:**
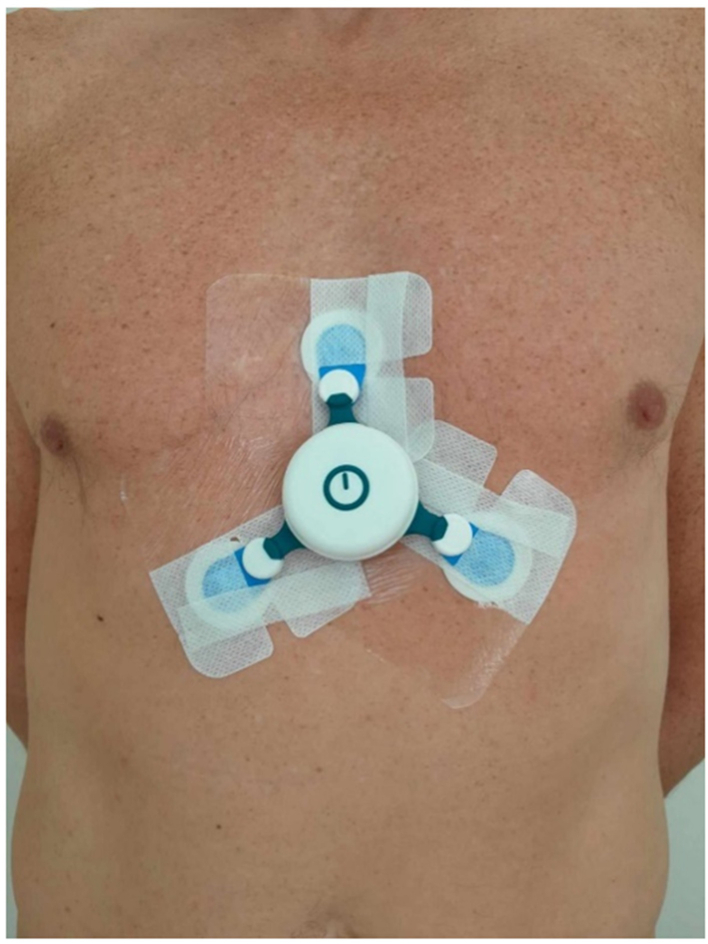
The ECG-device was placed with the two lower electrodes positioned on the lower part of the ribs and the upper electrode in the midline above. Adhesive tape was applied over and around the Ambu Blue Sensor P electrodes to secure the long-term positioning.

The wearable ECG patch device, C3 Monitor (Cortrium, Denmark), was used to record signals and stream real-time ECG data via Bluetooth to a smartphone (Google Pixel 4a, USA). The device was placed by EMU staff on the mid-chest, with two lower electrodes positioned on the lower ribs and an upper electrode in the midline above (Fig. 1). Ambu Blue Sensor P electrodes were used, and adhesive tape was applied over and around the electrodes to ensure long-term positioning and stable skin contact (Fig. 1). The device had a sampling frequency of 256 Hz and a battery life of up to four days with streaming enabled. Only the lead II signal (left to top electrode) was streamed to the smartphone for analysis. Lead II signal was used for analysis, based on our experience from previous phase-2 studies with the ePatch device,^10^^,^^11^ as well as initial testing of the C3 device in the first 10 patients of the current study. These tests demonstrated that lead II consistently provided the best signal-to-noise ratio for R-peak detection.

ECG signals, streamed via Bluetooth in batches of 16 samples to the smartphone, were processed in real-time to automatically detect R-peaks, noise, and to analyse HRV, using the ASSURE application (Automated Seizure Surveillance for Research in Epilepsy) developed by our team, using Android Studio version: 21·0·5 software. The R-peaks were detected using the Pan-Tompkins algorithm. We applied the HRV-based seizure detection algorithm, using CSI, which we developed in an exploratory study^10^ and subsequently validated in a phase-2 study,^11^ as described in detail in the following section.

**Fig. 2:**
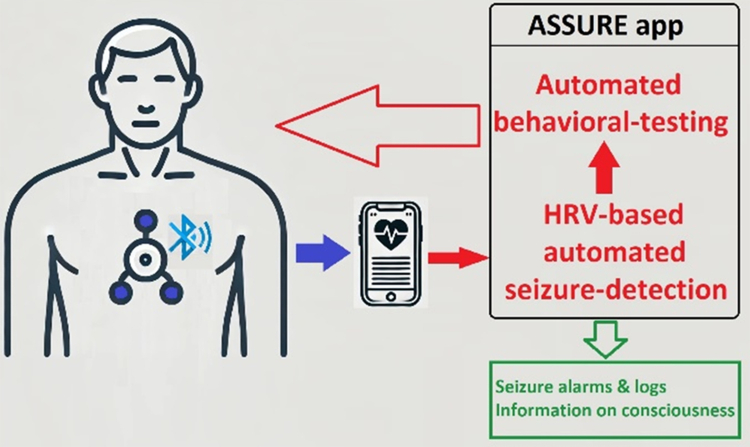
The wearable ECG device streams data continuously via Bluetooth to a smartphone. The ASSURE application monitors data quality, analyses Heart Rate Variability (HRV) in real time, and triggers a seizure alarm when a personalised detection threshold is surpassed. It then runs an automated behavioural-testing application to assess responsiveness and recall. Detected seizures and behavioural test results, including consciousness impairment, are logged.

When the seizure detection threshold was surpassed, the ASSURE app triggered a seizure alarm and logged the detection time. Fig. 2 shows an infographic illustrating the concept and information flow.

**Fig. 3:**
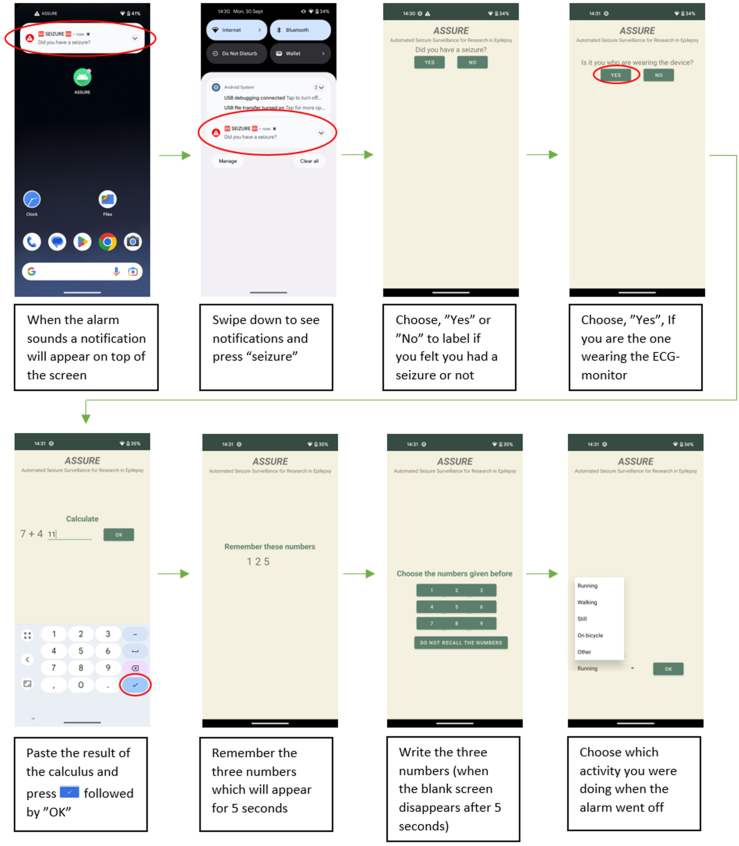
Screenshots from the smartphone, displayed during automated behavioural-testing for patients above 10 years old without intellectual disabilities.

Patients were instructed to respond to the alarm by answering questions displayed on the smartphone screen to assess their awareness and responsiveness and to confirm whether the detection was true or false (Fig. 3).

If patients were unable to respond, staff and caregivers were instructed on how to deactivate the alarm. The application displayed a question to identify the user (patient or caregiver) and prompted them to confirm or cancel the seizure-alarm. When used by the patient, the application presented a simple arithmetic task and a memory task (Fig. 3). During the study, to avoid waking patients during sleep, the alarm was automatically set to silent mode at night (11 pm–7 am) while the device was still logging seizure detections. Additionally, the alarm always remained in silent mode for patients under ten years old and those with intellectual disabilities, thus no behavioural-testing were done for these patients.

The ASSURE app continuously monitored signal quality and triggered a technical alarm (distinct from the seizure alarm) if the Bluetooth connection between the ECG device and smartphone was lost or if no R-peak was detected within 5 min (indicating very poor or no electrode-to-skin contact). Staff were trained to address these issues.

### Seizure detection algorithm

In our seizure detection device, we implemented the HRV-based algorithm initially developed in an exploratory study^6^ and later tested in a phase-2 study.^7^ This algorithm was the best-performing among 26 HRV-based seizure detection methods. Briefly, the Lorentz plot (Poincaré plot) was used to quantify heart rate variability (HRV). Beat-to-beat R-R interval variation is represented by four times the standard deviation (SD) of the transverse axis (T-value), while overall R-R interval variation is represented by four times the SD of the longitudinal axis (L-value), as described in detail below. The Cardiac Sympathetic Index (CSI) was calculated as L/T, and the Modified CSI (ModCSI) as L^2^/T. Both indices were derived using a sliding window of 100 R-R intervals to enable continuous estimation. The algorithm combines two HRV parameters: the Modified-CSI100-filtered X Slope and the CSI100 X Slope.^6^ The seizure detection threshold was individually calculated for each patient as 105% of the highest value of the two CSI parameters recorded during the baseline period (the first 24 h of monitoring). Only interictal data were used for baseline determination, excluding seizure epochs if any occurred during the baseline period, to ensure an accurate physiological baseline.

**Fig. 4:**
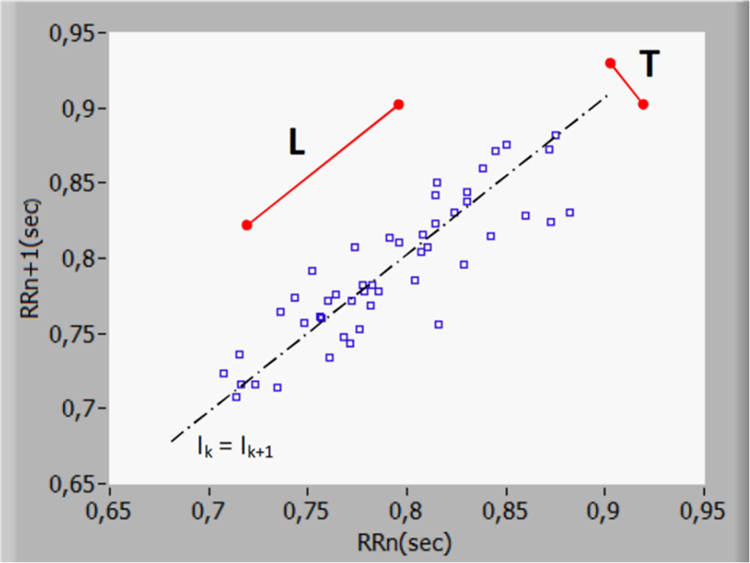
Lorenz plot with 50 R-R intervals. The transverse axis (T) reflects beat-to-beat variation (T = 4 ∗ SD1), while the longitudinal axis (L) reflects the overall fluctuations (L = 4 ∗ SD2).

Lorenz plot (or Poincare plot) is done by plotting each R-R interval time length (I_k_) against the following R-R interval time length (I_k_, I_k+1_) for a limited number of R-R intervals (k) (Fig. 4). Calculation of the standard deviations for the transverse direction (Sd1) which is perpendicular to the I_k_ = I_k+1_ line (1) and the longitudinal direction (Sd2) which is parallel to the I_k_ = I_k+1_ line (2) (Fig. 4) is done with the following equations:

(1) $$\text{Sd}1=\sigma \left(\frac{\sqrt{2}}{2}\left[\left({Ι}_{1}-{Ι}_{2}...{Ι}_{k}-{Ι}_{k+1}\right)\right]\right);k=\left\{100\right\}$$

(2) $$\text{Sd}2=\sigma \left(\frac{\sqrt{2}}{2}\left[\left({Ι}_{1}-{\;Ι}_{2}...{Ι}_{k}-{Ι}_{k+1}\right)\right]\right);k=\left\{100\right\}$$

where k represents the number of R-R intervals windowing (100).

The transverse length (T) is defined as four times the Sd1 and the longitudinal length (L) as four times the Sd2 (Fig. 4). An estimation of the sympathetic tonus from the Lorenz plot can then be given as respectively L/T for Cardiac Sympathetic Index (CSI) and L^2^/T for Modified Cardiac Sympathetic Index (ModCSI).

Cardiac Sympathetic Index (CSI), and Modified CSI (ModCSI), was calculated for each new heartbeat using 100 R-R intervals of sliding windowing with maximum overlapping. For the ModCSI100-filtered method a prefiltering with a seven R-R interval median filter of the tachogram was done (where the median time-length of the previous seven R-R intervals is found for each new heartbeat).

The Slope of tachogram is calculated as the absolute value of the slope of the prefiltered heart rate changes (=heart rate plotted against time). The slope of the heart rate changes was calculated using the Least Square method in the same 100 R-R windows as the CSI100 and ModCSI100_filtered. The Slope value for each datapoint was finally multiplied with both the CSI100 value and ModCSI100_filtered value for each datapoint to derive the final values of CSI100 X Slope and ModCSI100_filtered X Slope.

When the personalised seizure detection threshold of either ModCSI100-filtered X Slope or CSI100 X Slope is surpassed the seizure detection is registered by the algorithm, and the seizure alarm in the ASSURE-app would sound. If the detection is in the period within 1 min from seizure onset to seizure termination the detection is considered true positive. If not, it is considered a false positive. Whenever a detection is registered a 5-min refractory phase is imposed to avoid counting multiple detections for the same event. If a detection occurred during a seizure that was excluded, it neither counted as a seizure detection nor as a false alarm. Seizure detection latency was calculated from the seizure onset marked by the experts (authors SW and SB) to the point in time when the seizure was detected by the algorithm.

Additionally, a noise detector was embedded into the ASSURE application to eliminate excessively noisy periods caused by loose electrodes, which interfered with the ECG signal and made R-peak detection unreliable. The noise detector computes the root-mean-square (RMS) values of the ECG signal using 2-s windows. If the noise threshold (20 mV) is surpassed, both HRV parameters are set to zero to avoid false alarms due to poor signal quality. As mentioned earlier, longer periods of poor signal quality triggers an alert to notify personnel.

Data from the first ten enrolled patients were used to optimise the seizure alarm system. Consequently, these patients were excluded from the test dataset used to evaluate performance. The optimisation process involved several steps, including selecting the most suitable self-adhesive electrode patch type, determining the optimal electrode placement, testing the ASSURE app, and fixing bugs, adding smartphone alarms for Bluetooth disconnection and electrode contact loss, incorporating a noise detector, and training staff in the use of the app and alarms.

### Selection of patients with autonomic ictal changes

Previous studies have shown that HRV-based seizure detection is effective only in patients with significant autonomic changes.^10^^,^^11^ Therefore, we selected the patients eligible for HRV-based seizure detection: patients whose first recorded seizure exhibited an ictal heart rate increase of >50 beats/min during a 100 R-R interval period, based on previously published criteria.^10^^,^^11^ The rationale for this is, that heart rate, unlike HRV, can be easily recorded by a smartwatch, enabling the pre-selection of patients eligible for HRV-based seizure detection in a home environment.

### Performance evaluation

The first ten patients, assigned for system optimisation, were excluded from the analysis. All subsequently recruited consecutive patients were included in the test dataset to evaluate device performance. The diagnostic reference standard (“gold standard”) was derived from long-term video-EEG recordings in the EMU. Experts (board-certified physicians with over 10 years of experience) identified seizure onset as the first detectable electroencephalographic or clinical sign (whichever occurred first) and seizure termination as the last observed sign (whichever occurred last). The experts were blinded to the HRV analysis. Seizures without clinical correlates (purely electrographic) and those shorter than 20 s were excluded (seizure alarms occurring during these seizures neither counted as true or false positives). For seizures occurring in clusters with intervals of less than 30 min, the start of the first seizure was used to define the cluster period.

For each patient, the first 24 h of recording were used to establish an individual baseline (detection threshold), and the remaining data were used to evaluate device performance. The first seizure was used to classify the patient as with or without ictal autonomic changes (threshold: 50 bpm ictal change). Automated detections logged by the device were compared to the reference standard. Detections within 1 min before or after the marked seizure onset and offset were classified as true positives. Multiple detections occurring within a 5-min window were counted as a single alarm. Detections outside seizure periods were considered false positives.

### Statistics

The primary outcome was the performance of the seizure detection device in patients experiencing ictal autonomic changes. This was assessed using the following metrics:

- Sensitivity: True positive/(True positive + False negative).
- False alarm rate: Number of false detections per 24 h.
- Device deficiency time: Periods of electrode-skin contact loss. This was subtracted from the total evaluated time.
- Signal loss: Periods of signal loss due to Bluetooth disconnection or battery discharge.

We report overall sensitivity, calculated from all recorded seizures,^12^^,^^17^ as well as per-patient statistics, where each patient is analysed independently before summary statistics are calculated, as previously described.^18^

The secondary outcome was the performance of the application for automated testing of patient behaviour during seizures. This was evaluated based on its ability to correct false detections and provide accurate information on impairment of consciousness during seizures.

### Role of funders

The funding source had no role in study design, data collection, data analysis, data interpretation, manuscript writing, or the decision to submit for publication.

## Results

Of the 111 recruited patients, the first 10 were assigned to system optimisation tasks. Among the remaining 101 patients, 36 experienced electroclinical seizures (22 male; median age 34 years; range: 6–67 years, IQR: 23·5–48·5 years), of whom 17 (47·2%) met the eligibility criteria for ictal autonomic changes (13 male; median age 32 years; range: 6–67 years, IQR: 24–40 years; 13 monitored at Aarhus University Hospital and 4 at the Danish Epilepsy Center). The eligible patients collectively had 42 seizures, including 23 focal seizures (14 with impaired consciousness and 9 without) and 19 tonic-clonic seizures (one generalised and 18 focal-to-bilateral). All patients who experienced tonic-clonic seizures met the eligibility criteria for ictal autonomic changes. The median seizure duration was 101 s (IQR: 63–122 s), based on the gold standard. The total streamed recording time during the test period with the enabled seizure detection device was 3530 h in total and 880 h for the eligible patients (median per patient: 56·5 h, range: 6·5–118 h).

The overall sensitivity across all 42 seizures was 90·5% (95% CI: 77·4–97·3%). The median sensitivity per patient was 100% (95% CI: 100–100%; range: 50–100%), with all seizures detected in 14 patients. Of the 20 seizures that occurred during the daytime, one was missed, while three of the 22 seizures that occurred at night were missed. Of the 23 focal seizures which did not evolve to bilateral tonic-clonic seizures, 19 were detected (sensitivity: 82·6%, 95% CI: 61·2–95·1%; median sensitivity: 100%), while all 19 bilateral tonic-clonic seizures were detected (sensitivity: 100%, 95% CI: 82·4–100%).

The mean false alarm rate (FAR) in the eligible patients was 2·5 per 24 h (median FAR per patient: 1·1 per 24 h; 95% CI: 0–2·8). During sleep, the mean FAR was 0·5 per night (median FAR: 0 per night; 95% CI: 0–1). ASSURE automatically pauses alarms for 107-RR intervals following seizure detection to prevent duplicate alarms during the same seizure event. If we remove the 5-min refractory, resulting in multiple false alarms within a 5-min window, the number of false positives increases from 90 to 102 (FAR from 2·5 to 2·8 per 24 h), and 14 duplicate alarms occur during seizures. During the entire monitoring period with enabled seizure detection in all patients (3530 h), the mean false alarm rate (FAR) was 1·9 per 24 h (median: 1·1 per 24 h, 95% CI: 0·4–1·1 per 24 h).

The median detection latency was 28 s (range: 15–278 s). Individual metrics, demographic, and clinical data for all eligible patients are shown in Supplementary Table s1.

For the 19 patients who were not eligible for HRV-based seizure detection, based on the pre-defined criterion, the algorithm detected only 16·4% (95% CI: 8·8–27·0%) of the 73 seizures (all focal). The mean FAR in the non-eligible patients was 1·5 per 24 h (median: 1·5 per 24 h; 95% CI: 0–2·2).

Signal loss, due to Bluetooth disconnection or battery discharge, accounted for 4·5% of the total monitoring time (median per patient: 0·3%; 95% CI: 0–3·2%) and device deficiency time, due to poor electrode-skin contact, accounted for 1·8% of the total streamed time in eligible patients (median per patient: 0·5%; 95% CI: 0–2·2%). Detailed information for each patient (including seizure onset zone, anti-seizure medications, tapering status, signal loss, technical warnings and clinical vs EEG-seizure onset) is provided in Supplementary Table s2. The primary cause was that eight technical warnings were not addressed according to the study protocol, resulting in three periods of signal loss exceeding 15 h each. According to the protocol, a new recording should have been initiated by personnel upon receiving a technical warning. A total of 31 technical warnings occurred, averaging 0·58 per 24 h.

The STARD flowchart is shown in Supplementary Figure s1.

During periods when automated behavioural-testing was enabled on the smartphone, 73 automated detections were recorded: 16 true alarms (seizures) and 57 false alarms. Patients or caregivers used the smartphone application as instructed during 62 events (85% of the alarms), comprising 15 seizures and 47 false alarms. The application accurately cancelled all 47 false alarms and correctly confirmed all 15 epileptic seizures. No seizures were cancelled, and no false alarms were misidentified as seizures. Consequently, the combination of automated seizure detection and behavioural-testing achieved 100% adjusted accuracy in seizure quantification—i.e., it correctly identified 15 out of 15 seizures among 62 events (true alarms (seizures) and false alarms). Additionally, the application correctly identified impaired consciousness in all 9 seizures where it occurred.

Of the 101 enrolled patients, 16 requested the removal of the wearable device before completing the study: 14 due to skin irritation, one due to multiple technical alarms, and one due to anxiety. Two of these 16 patients were eligible for HRV-based seizure detection. Their data were analysed until withdrawal. The time until withdrawal ranged from 4 h to 85 h (median: 35·5 h; IQR: 19–52 h).

## Discussion

In this phase 3, prospective, blinded, multicentre study, we evaluated real-time automated seizure detection based on a predefined HRV algorithm using a wearable ECG device connected to a smartphone. Eligibility was assessed based on the first recorded seizure (to confirm autonomic ictal changes), and the individual detection threshold was determined using the first 24 h of recordings. Nearly half of the patients (47·2%) exhibited autonomic ictal changes and were thus eligible for HRV-based automated seizure detection. All seizures were detected in 82·3% of the eligible patients. Across 42 seizures in eligible patients, the overall sensitivity was 90·5%. Bilateral tonic-clonic seizures were detected in all patients, while sensitivity for other, focal seizure types was 82·6%.

The median false alarm rate was 1·1/24 h (zero during the night). A small subgroup of patients (n = 4) accounted for most false alarms: the four recordings with the highest number of false alarms contributed 65 of the 90 false alarms (72%). Notably, two of these patients did not complete the exercise bike task during the baseline 24-h period, which may have led to an individual detection threshold that was set too low for these patients. Excluding these two patients gives a mean FAR of 1·66/24 h (median per patient: 0·6 per 24 h). Ideally, all patients would have completed all three baseline tasks to ensure optimal estimation of individual detection thresholds. However, requiring full participation in these tasks would have been ethically inappropriate, as it may place undue burden on some patients. Instead, we opted to keep participation optional, reflecting a more realistic, real-world scenario where not all patients may be able to complete all baseline tasks. We reviewed the video recordings to identify the major sources of false detections. Of the false detections, 20 occurred during movement or position changes (e.g., standing up or sitting down), 17 during transitions between wake and sleep states, 16 in the bathroom or while going to the bathroom, and 12 in the postictal state. For the remaining false detections, we could not identify any clear behavioural correlates. Seizure detection based on modified CSI index has much lower FAR than tachycardia-based seizure detection, because this HRV measure is more specific for ictal changes than tachycardia, which occurs both during seizures and physical activity.^8^^,^^9^ In this context it is important to point out that patients in our study had a large degree of mobility in the EMU. We used wireless amplifiers, and the EMU was designed specifically for this purpose, allowing patients to move freely within their room and within the EMU which at the Danish Epilepsy Centre included a living room, a dining room, a space for physical exercise (treadmill, indoors bike), playground for children, and a patio with rubber floor.^13^

Automated behavioural-testing, triggered by seizure detection and conducted via the smartphone, correctly cancelled all false detections. Consequently, the combination of automated seizure detection and behavioural-testing achieved 100% accurate seizure quantification. Furthermore, the application correctly identified impaired consciousness in all seizures where it occurred.

Eliminating false alarms is essential for reliable seizure quantification and remains a top priority for patients and caregivers.^19^ In previous retrospective (phase 2) studies,^20^^,^^21^ we sought to address this challenge using a personalised detection algorithm that continuously adapts throughout the recording, to improve performance. Although it significantly reduced the FAR to 0·22 per 24 h, it did not eliminate all false detections and led to a decrease in sensitivity (71·4%). Therefore, in this phase 3 study, we opted to use automated behavioural-testing to address false alarms. This approach proved successful and also provided valuable additional information about the seizure, such as impairment of consciousness.

Previously, high-accuracy automated detection using wearable devices in phase 3 validation studies has only been achieved for tonic-clonic and absence seizures, achieving sensitivity between 90 and 100%.^1^, ^2^, ^3^ In this phase 3 study, we demonstrated that all tonic-clonic seizures were accurately detected, which is crucial for safety indications, as this seizure type is associated with high morbidity and mortality. Additionally, 82·6% of focal seizures that did not develop into bilateral tonic clonic seizures were detected using the wearable ECG device. To the best of our knowledge, this is the first phase 3 validation study of a non-invasive, wearable device to successfully detect both of these seizure types. Our study is the first to report the use of automated behavioural testing triggered by automated seizure detection in a wearable device, assessing both responsiveness and awareness (recall). While a previous study demonstrated the feasibility of automated testing of responsiveness in patients with absence seizures using a wearable device,^22^ and another study implemented automated behavioural testing in a seizure detection system based on full-array EEG.^17^ Our approach is novel in its combination of non-invasive wearable device and cognitive assessment. Combining automated seizure detection based on HRV with automated behavioural-testing eliminated all false detections, which is essential for objective seizure quantification.

We would like to highlight several limitations. The number of eligible patients in our study was relatively small (n = 17). In our study, 16% of enrolled patients removed the wearable device before completing the monitoring period, primarily due to skin irritation caused by the self-adhesive electrode patches. This withdrawal rate was significantly higher than in our previous (phase 2) studies using a different patch-ECG device (ePatch), where only 2·0% of patients discontinued participation.^10^^,^^11^ The skin irritation appeared to result from the bulky design of the device used in this study (Cortrium C3), which required additional adhesive tape to secure the electrode patches and maintain proper ECG signals. Technical failures, including signal loss (median per patient: 0·3% of time) and poor electrode-skin contact (median per patient: 0·5% of time), represent important limitations. Consequently, the feasibility of ultralong-term monitoring with this device is uncertain. More patient-friendly and technically stable methods, such as garment-integrated electrodes or subcutaneous ECG recording, are likely better suited for prolonged ECG monitoring.^23^^,^^24^ A recent phase 2 study using garment-integrated electrodes in the Hexoskin shirt, which incorporates ECG and accelerometry, showed promising results with an 84·4% detection sensitivity for focal to bilateral tonic clonic seizures and a mean false alarm rate of 0·55 per 24 h.^23^ However, this study did not address the detection of focal seizures that did not develop into bilateral tonic clonic seizures.^23^ In our recent, proof-of-concept study, a subcutaneously implanted ECG device detected 92·6% of 54 focal seizures that did not develop into bilateral tonic clonic seizures using the same HRV algorithm employed in this phase 3 study.^24^ Notably, all six patients completed ultralong-term monitoring with the subcutaneous device - for up to 8 months.^24^ An additional advantage of subcutaneously implanted ECG devices over surface devices is the stability of electrode contact, eliminating the need for daily electrode changes and frequent adjustments triggered by technical alarms indicating poor contact with surface devices. The HRV-based seizure detection algorithm relies on the analysis of a sliding window of 100 RR intervals, and a minimal signal timeframe of 20 s is required to trigger a seizure.^8^, ^9^, ^10^, ^11^ While a 10-s timeframe is commonly used for electrographic seizures, this is somewhat arbitrary, reflecting historical practices when EEG data were typically reviewed in 10-s screen segments. However, our algorithm is designed for use in patients with epilepsy, focussing on clinical rather than purely electrographic seizures, which distinguishes it from approaches used in critically ill patients. Notably, our 20-s threshold reflects a clinical focus, as focal or autonomic seizures shorter than 20 s are generally less impactful on patients' quality of life.

It is important to note that not all patients with epilepsy are eligible for HRV-based seizure detection. Eligibility is limited to patients with autonomic nervous system involvement during seizures. In our study, nearly half of the consecutively recruited patients met this criterion. A heart rate increase of 50 bpm during seizures is a reliable indicator of autonomic involvement. This can be easily assessed using commercially available smartwatches, which reliably detect heart rate. In contrast, HRV measurement requires higher precision of R-peak detection, achievable only with ECG devices. The study population was representative of the broader Danish epilepsy community; however, subgroup analyses based on gender and ethnicity were not performed. Future studies should explore whether seizure detection accuracy varies across demographic groups.

In conclusion, this phase 3 study demonstrated high accuracy of automated seizure detection and behavioural testing using a wearable ECG device connected to a smartphone. The system achieved performance sufficient for both safety indications and objective seizure quantification in patients with ictal autonomic changes. However, the ultralong-term stability of the wearable ECG patch device remains uncertain. Implementing this algorithm in subcutaneous ECG devices is likely to address this challenge.

## Contributors

J.J. and S.B. conceived the seizure detection system, designed the study, collected, analysed, and interpreted data, and drafted the manuscript.

O.A.P, S.F., S.R.W., and P.J. designed the smartphone application, contributed to the interpretation, and edited the manuscript.

J.C., S.A.L., and S.W. collected and interpreted the data and edited the manuscript.

The first and last authors had full access to all the data in the study and take responsibility for the integrity of the data and the accuracy of the data analysis.

All authors read and approved the final version of the manuscript.

## Data sharing statement

Our HRV algorithm used in this study is open source, for non-commercial use, as described in the Methods section. Anonymised individual clinical trial participant-level data (IPD) will be shared upon request. ECG signals recorded with the wearable device, seizure time-points according to the diagnostic gold standard, demographics (age, gender) and additional, related documents will be available upon request. As the dataset is de-identified, there is no need for consent from the participants. Data will be available upon request, for 10 years from the publication, for scientific non-commercial use.

## Declaration of interests

We have no conflict of interest to report related to this work.

## Appendix A Supplementary data

Supplementary Tables and Figure

Clinical Research Plan
