# Supplementary material for: Seizure detection using wearable electrocardiogram connected to a smartphone: a phase 3 clinical validation study
Source: eBioMedicine. 2025 Sep 29;120:105952. doi: 10.1016/j.ebiom.2025.105952 (PMC12516532; doi:10.1016/j.ebiom.2025.105952)
Supplement: Supplementary Tables and Figure [file mmc1.docx]

## **Supplementary Materials**

**Supplementary Table s1**

## Individual data of the eligible patients page 2

**Supplementary Table s2**

## Individual data of the eligible patients page 3

**Supplementary Figure s1**

The STARD flowchart of the study page 4

##

*Table s1*

**Individual data of the eligible patients: demographics, seizures, seizure types, performed tests, false alarm rates and detection sensitivity.**

| **Patient number** | **Gender** | | **Age** | **Seizures recorded** | **Seizure type** | **Impaired awareness** | **Performed tests** | **False alarm rate**  **(per 24h)** | **Test data (hours)** | **Number of false positives** | **Detection sensitivity (%)** |
| --- | --- | --- | --- | --- | --- | --- | --- | --- | --- | --- | --- |
| 1 | | M | 46 | 2 | Focal (Hypermotor) | No | E,C,H | 0.0 | 21.5 | 0 | 100 |
| 2 | | M | 35 | 3 | 2 Focal, 1 FBTC | Yes | E | 1.1 | 65 | 3 | 100 |
| 3 | | M | 24 | 1 | FBTC | Yes | E,C,H | 4.6 | 62 | 12 | 100 |
| 4 | | M | 10 | 2 | 1 Focal (Tonic), 1 FBTC | Yes | E | 0.0 | 25 | 0 | 100 |
| 5 | | F | 24 | 1 | FBTC | Yes | E,C,H | 0.0 | 24.5 | 0 | 100 |
| 6 | | F | 20 | 1 | GTC | Yes | C,H | 3.4 | 100 | 14 | 100 |
| 7 | | F | 21 | 1 | Focal | Yes | E,C | 0.5 | 50.5 | 1 | 100 |
| 8 | | M | 33 | 7 | Focal | No | E | 2.8 | 34.5 | 4 | 86 |
| 9 | | M | 34 | 1 | FBTC | Yes | E | 1.7 | 27.5 | 2 | 100 |
| 10 | | F | 66 | 1 | FBTC | Yes | E | 2.8 | 68.5 | 8 | 100 |
| 11 | | M | 25 | 3 | 1 Focal, 2 FBTC | Yes | N | 9.0 | 69 | 26 | 100 |
| 12 | | M | 27 | 4 | FBTC | Yes | E,C | 1.1 | 68.5 | 3 | 100 |
| 13 | | M | 44 | 1 | Focal | Yes | E | 0.0 | 6.5 | 0 | 100 |
| 14 | | M | 40 | 1 | FBTC | Yes | E,C,H | 0.4 | 56.5 | 1 | 100 |
| 15 | | M | 6 | 5 | Focal | Yes | E | 0.0 | 16.5 | 0 | 60 |
| 16 | | M | 32 | 2 | Focal | Yes | E,C,H | 4.7 | 66.5 | 13 | 50 |
| 17 | | M | 67 | 6 | 1 Focal, 5 FBTC | Yes | E,C,H | 0.6 | 118 | 3 | 100 |

Patient 3 and 11, were two admissions to the EMU, 6 months apart, of the same patient (separate baseline periods included separate detection thresholds were conducted).

Abbreviations: E = Exercise test, C = Cognitive test, H= Horror movie viewing, N = None, FBTC = Focal to bilateral tonic-clonic, GTC = generalized tonic-clonic.

The detection threshold for each admission was based on the initial 24-hour baseline recording, regardless of whether the patient completed any of the optional baseline tasks. While we encouraged patients to complete these tasks, participation was not required

*Table s2*

**Additional data of the eligible patients: Seizure onset zone, ASM at the time of admission, tapering off ASM, Time of signal loss due to uncharged device or loss of Bluetooth connection, Number of technical warnings, Number of technical warnings not managed according to the protocol (No-signal, re-start within 15 minutes).**

| **Patient number** | **Seizure onset zone** | **ASM** | **Tapering** | **Signal loss (hours)** | **Technical warnings** | **Technical warnings not managed appropriately** |
| --- | --- | --- | --- | --- | --- | --- |
| 1 | Frontal lobe | Lamictal, Keppra | No | 1.5 | 2 | 1 |
| 2 | Right Temporal | Lamictal, Topimax, Levetiracetam, Diazepam | Yes | 0 | 0 | 0 |
| 3 | FBTC Left hemisphere (lobe uncertain) | Lamictal, Levetiracetam | Yes | 2.5 | 1 | 1 |
| 4 | Multi focal (Lennox-Gastaut syndrome) | DNA | DNA | 16 | 1 | 1 |
| 5 | Right Temporal | Duloxetin | No | 0.9 | 2 | 1 |
| 6 | GTC | Deprakine Retard, Lamotrigin, Zonegran | No | 16 | 2 | 1 |
| 7 | Left Temporal | Levetiracetam, Lamictal | Yes | 20 | 6 | 2 |
| 8 | Right Temporal | Levetiracetam, Lamotrigin | No | 0 | 0 | 0 |
| 9 | Right Temporal | Tegretol, Fycompa | Yes | 0 | 1 | 0 |
| 10 | Left Frontal | Levetiracetam, Lamotrigin, Lacosamid | Yes | 0 | 0 | 0 |
| 11 | Left posterior temporo-occipital | Lamotrigin, Levetiracetam | Yes | 0 | 0 | 0 |
| 12 | Temporo-frontal (lateralizing uncertain) | Fycompa, Lamotrigin | No | 0.75 | 0 | 0 |
| 13 | Left Temporal | Tegretol, Fycompa | Yes | 0 | 0 | 0 |
| 14 | Right Temporal | Brivaracetam, Oxcarbazepin | Yes | 3.5 | 9 | 1 |
| 15 | Right Frontotemporal | Lacosamid, Valproinsyre | No | 0.1 | 2 | 0 |
| 16 | Uncertain | Levetiracetam, Carbamazepin | No | 0.3 | 5 | 0 |
| 17 | Left Temporal | Vimpat, Lamotrigin, Rivotril | Yes | 0 | 0 | 0 |

Patient 3 and 11, were two admissions to the EMU, 6 months apart, of the same patient (separate baseline periods included separate detection thresholds were conducted).

Abbreviations: GTC = generalized tonic-clonic, DNA = Data not available, ASM = Anti-seizure medicine.

Additional information regarding eligible patients: No cardiac comorbidities or arrhythmias were observed. Of the 38 detected seizures, two showed a significant discrepancy between EEG and clinical onset times: In patient 6 (GTC), the EEG onset preceded the clinical onset by 130 seconds. The detection occurred 144 seconds after the EEG onset but only 14 seconds after the clinical onset. In patient 10 (FBTCS evolving into status epilepticus), the EEG onset preceded the clinical onset by 239 seconds, with the detection occurring 278 seconds after the EEG onset and 39 seconds after the clinical onset. In one patient with five seizures, the difference between EEG and clinical onset times ranged from 10 to 35 seconds, with EEG preceding clinical onset in two cases and clinical onset preceding EEG in three cases. However, there was no consistent correlation between detection time and onset type in these seizures. For all other seizures across all patients, the difference between EEG and clinical onset times was less than 10 seconds, suggesting a closer correlation between detection times and clinical onset overall.

As only 3 of the 17 patients did not have 100% detection sensitivity, no reliable statistical analysis could be done to stratify for ASM influence of seizure detection sensitivity.

*Figure s1*

**The STARD flowchart of the study**
