# Supplementary material for: Seizure detection using wearable electrocardiogram connected to a smartphone: a phase 3 clinical validation study
Source: eBioMedicine. 2025 Sep 29;120:105952. doi: 10.1016/j.ebiom.2025.105952 (PMC12516532; doi:10.1016/j.ebiom.2025.105952)
Supplement: Clinical Research Plan [file mmc2.pdf]

Seizure alarm with wearable electrocardiogram device for people with epilepsy

Approximately 55,000 persons in Denmark suffers from epilepsy[1] and about 30% of these never achieve seizure-freedom despite different treatment techniques such as medication, diets and surgery. For these patients, seizures are unpredictable and can arise without any warning and can produce injury, psychiatric illness, cognitive impairment and reduced independence. Unattended seizures are highly dangerous leading to serious injuries and markedly increase the risk of sudden unexpected death in epilepsy patients[2]. Also, therapeutic decisions are based on seizure frequency self-reported by the patients. However, these are largely unreliable, since patients cannot notice or remember more than half of their seizures[3]. In recent studies we have shown that analysis of heart rate variability (HRV), measured with electrocardiogram (ECG) can detect seizures in patients with epilepsy[4–7].

Aims and perspectives

The overall aim of this study is to investigate if our HRV-based seizure detection algorithm[6] measured by small, wearable ECG-devices, is reliable as a seizure alarm system.

Our hypotheses is:

> *Heart rate variability algorithms implemented into a smartphone application with signals received from a small, non-invasive, wearable and wireless ECG-device can reliably detect seizures real-time, and send seizure-alarms.*

We will use the non-invasive, wearable CE-marked ECG-device C3+ from Cortrium to record the ECG. The C3+ ECG-device has the opportunity to stream the recorded signals directly to a smartphone via Bluetooth and such make us able to use our validated seizure detection algorithm in the seizure detection app real-time. **The seizure detection app is a prototype developed by our group at Aarhus University and does not have a CE-mark and the purpose with this study is not to obtain CE-mark of the app (article 82).**

Currently there are no seizure detection alarms available that can detect of focal seizures, with impaired consciousness[8,9], so the patients with these seizure type have no way of alerting caretakers or family when a seizure is occurring. The proposed wearable epileptic seizure alarm device will enable caregivers and family members to take the necessary precautions to decrease morbidity and mortality related to seizures. In addition, the seizure alarms will provide objective data on the patients´ seizure burden. The seizure alarm will thus contribute to a much more secure life with better job-, social- and mobile opportunities for the untreatable epileptic patients, as well as better data for fine-tuning therapy. In severe epileptic cases the alarm system may even save lives, as it is well established

that unattended seizures significantly increases the risk of the patient dying of sudden unexpected death in epilepsy[2].

Background

Electroencephalogram (EEG) is the most commonly used investigation method of diagnosis of epilepsy and most of the research for automatic detection of seizures has been based on this method, the results have however been inconsistent[10]. Multichannel EEG is furthermore a relatively inconvenient and uncomfortable method of measuring and it is almost impossible for patients to apply and maintain an EEG, making it unsuitable as a portable seizure alarm. Thus, there are several good reasons to investigate other, easier measurable, physiological parameters that are potential biomarkers of epileptic seizures, like the heart rhythm changes measured with wearable ECG.

A recent systematic review of the published literature concluded that there is evidence only for detection of generalized tonic-clonic seizures, using wearable devices, and that detection of focal seizures, with impaired consciousness remains an unresolved challenge[8,9]. The current commercially available wearable seizure detection devices are only able to detect generalized tonic-clonic seizures. These devices are either using accelerometry (ACC) (Epi-Care from Danish Care[a]) or ACC in combination with photoplethysmography (NightWatch from NightWatch[b] and Embrace from Empatica[c]). However, currently, no approved seizure detection devices are able to detect focal seizures, which is a considered a major issue as the majority of epileptic seizure are focal seizures[8,9].

Heart rate variability (HRV) is calculated by making a frequency analysis of the heart rate measured by electrocardiogram (ECG). Different analyses have shown that patients with temporal lobe and frontal lobe epilepsy have an overall lower HRV than healthy individuals, which suggests a dysfunction of the autonomic nervous system[11–16]. These studies, however, have not evaluated if an online seizure detection is possible. A few of the published studies had specific focus on seizure detection, but they had not been able to test this in an online dedicated wearable ECG-device[8]. In recent studies, we have shown that HRV parameters, measured with a potable ECG-device analysis can detect both generalized and focal seizures for patients with epilepsy (Jeppesen et al., Epilepsia 2019, Jeppesen et al. Epilepsia 2020)[6,7]. By use of the HRV Lorenz plot method we created an algorithm, based on a modified measure of an instant cardiac sympathetic index that positively detected seizures for patients with epilepsy[4,6,7]. In these research studies, the portable CE-marked ECG-device ePatch were used to record the ECG of the patients while the patients were hospitalized at the long-term video-EEG monitoring unit for diagnostic reasons or presurgical evaluation, which

---

[a] https://www.danishcare.dk/

[b] https://www.nightwatchepilepsy.com/

[c] https://www.empatica.com/

is the same approach we will use in this submitted study. The ePatch device could store the ECG-data of the patients which we used for optimizing the seizure detection algorithm in 100 recruited patients[6] and later validated in 43 patients[7]. 23 of the recruited patients were children (age range was 4-17 years old).

The results of the previous studies where the algorithm was developed was highly positive. In the study optimizing the algorithm[6] the results were as follows: 100 consecutive patients and analyzed 126 seizures were analyzed (108 nonconvulsive and 18 convulsive) from 43 patients who had seizures during monitoring. The algorithm identified 53.5% of the patients with seizures as responders. Among responders, detection sensitivity was 93.1% (95% CI: 86.6%-99.6%) for all seizures and 90.5% (95% CI: 77.4%-97.3%) for nonconvulsive seizures. False alarm rate was 1.0/24 h (0.11/night). Median seizure detection latency was 30 s. Typically, patients with prominent autonomic nervous system changes were responders: An ictal change of >50 heartbeats per minute predicted who would be responder with a positive predictive value of 87% and a negative predictive value of 90%.

, and in the validation study[7] the results were likewise very confident:

Responders were defined as the patients who had a >50 beats/min ictal change in heart rate. Eleven of the 19 included patients with seizures (57.9%) fulfilled this criterion. In this group, the algorithm detected 20 of the 23 seizures (sensitivity: 87.0%). The algorithm detected all but one of the 10 recorded convulsive seizures and all of the 8 focal impaired awareness seizures, and it missed 2 of the 4 focal aware seizures. The median sensitivity per patient was 100% (in nine patients all seizures were detected). The false alarm rate was 0.9/24 h (0.22/night).

Both adults and children were recruited in the studies as it is of outmost importance that a seizure detection alarm can be used for both adult and children and it is known that the heart rate variability changes can be different for adult and children. Also, epileptic seizures are very heterogeneous in their nature and some types of seizure are only seen in children, therefore it is very important to verify that these seizure types can also be detected using the seizure detection algorithm.

In the submitted study we will use the same approach as in the previous studies except instead of the CE-marked ECG-device ePatch we use another, similar, wearable device the CE-marked ECG-device C3+ from Cortrium. The devices are very similar (non-invasive, wireless, wearable patch CE-marked ECG-devices) except that the C3+ from Cortrium has the opportunity to stream the recorded signals directly to a smartphone via Bluetooth and such make us able to use our validated seizure detection algorithm in the seizure detection app real-time.

Our hypothesis is that more than 40% of the enrolled patients will be positive responders and that these patients will have a seizure detection sensitivity of more than 80% of seizure with a false alarm rate of less than 1,2 pr. day using our seizure detection system

In order to test our hypotheses, we will:
Automatize the HRV-based seizure detection algorithm and embed it directly into a smartphone that receives a continuous Bluetooth stream from the wearable, non-invasive wireless ECG-patch, and validate it in a phase-3 clinical study[17].
All recordings will be done in the long-term video/EEG monitoring at Aarhus University Hospital or Danish Epilepsy Center. We use the portable, wireless CE-marked (IIa) ECG-device (C3+ Holter Monitor patch)[18]. An NDA agreement has been made with the manufacturer and developing engineering group of the C3+ Holter Monitor patch ECG-device from Cortrium to gain access to the raw ECG-data, which has been streamed to the smartphone via Bluetooth. The company (Cortrium) will not, at any time, have access to any of the data recorded. The C3 Holter Monitor patch device is a small ECG-device that can be attached to the chest and be worn for up to 24 hours without change of battery. Furthermore, the device can transmit the ECG-data online via Bluetooth to a smartphone where our automatic seizure detection algorithm is embedded.

Methods & Research Plan
The ICD-10 code under investigation in the study is ICD-40 Epilepsy.
150 patients will be recruited. The two long term video/EEG monitoring at Aarhus University Hospital and Danish Epilepsy Center will contribute equally with approximately 75 patients recruited from each place. Approximately 1/3 of the recruited patients will be children (3-17 years old).
The study will strictly follow the guidelines of a phase-3 clinical study for testing and validation of seizure detection devices as it has been outlined in "Standards for testing and clinical validation of seizure detection devices". This entails: 1) a minimum number of 20 patients with seizure, 2) at least 30 seizures, 3) a dedicated device, 4) continues measurement, 5) multicenter study, 6) a predefined algorithm and cutoff values, 7) real-time seizure detection, 8) reference standard being video-EEG recordings. Both the adult and the children sub-group will individually fulfill the guidelines of a phase-3 clinical study.

Patients

Inclusion criteria:

- Patients enrolled for long term video/EEG monitoring at Aarhus University Hospital or Danish Epilepsy Center.
- Age above 3 years old.
- Diagnosis of probable focal or generalized epilepsy.

Exclusion criteria:

- Pregnant women will be excluded in the study, due to foreseeable noise on the ECG-recording.
- Incompetent adults (inhabil).
- Patients with known heart rate decreases or abnormalities which are known to cause heart rate disturbances.
- Patients with pacemakers or any implanted electronic device.

The participant-information (bilag 2) will be sent to the electable patients or guardians, so they on behave of that and the oral information given by the clinical responsible of the project, can decide whether or not to participate in the project. It is clarified in the written information that the patients can obtain further information of the project by contacting the clinical responsible of the project. All enrolled patients are covered by the Patient Compensation Association. The guidelines of oral information and written consent will be given as described in Danish below:

Retningslinjer for mundtlig deltagerinformation og indhentning af samtykke

Rekruttering foregår ved at patienter tilknyttet Neurologisk Afdeling på Århus Universitetssygehus eller Epilepsihospitalet i Dianalund, og som opfylder kriterierne for inklusion, får tilsendt eller tildelt brevet: Deltagerinformation (Deltagelse i et videnskabeligt studie). I brevet beskrives forsøgets formål og betydning for samfundet, og forsøgets risici og konsekvenser for deltager. Brevet vedhæftes forskningsministeriets informationsbrochure "Forsøgspersoners rettigheder i et sundhedsvidenskabeligt forskningsprojekt", som patienterne vil blive bedt om at læse grundigt. Ligeledes står der i brevet at vedkommende har ret til en bisidder ved informationsmødet, samt at enhver deltagelse i forsøget kan afbrydes fra deltagerens side på enhver tidspunkt, uden behov for begrundelse, og uden at det påvirker patientens nuværende eller fremtidige behandlingstilbud.

I brevet angives navn, adresse og telefon nr. på kontakt personer for eventuelle spørgsmål. Når patienterne har vist interesse i at deltage i forsøget, aftales der et informationsmøde med dem, hvor den klinisk ansvarlige informerer dem mundtligt, om forsøgets formål, risici og konsekvenser for de deltagende. Når patienter efter betænkningstid på mindst 24 timer beslutter sig for at deltage, bedes de om at underskrive den skriftlige samtykkeerklæring.

For informationsmødet findes følgende retningslinjer:

Samtalen skal være planlagt og kunne foregå uforstyrret, dvs. i et lokale som er reserveret til det formål.

Der skal være tilstrækkeligt tid til at gennemgå den skriftlige information, lytte til den mundtlige information og stille spørgsmål. Der gives en forståelig fremstilling af forskningsprojektet uden brug af tekniske eller værdiladede vendinger.

Samtykke bedes givet snarest efter betænkningstiden, der som udgangspunkt skal være mindst et døgn. Denne kan foregå ved ankomst til planlagt video/EEG overvågning. Informationen til forsøgsdeltageren gives af den kliniske ansvarlige læge på det pågældende site.

Informationen skal fremstilles på en enkel og let forståelig facon, og skal tage hensyn til individets forudsætninger for forståelse af materialet.

Der skal informeres om projektets formål og fremgangsmåde, samt at der kan være uforudsigelige risici og belastninger knyttet til deltagelse i projektet, hvilket ligeledes skal gøres under gennemførelsen, hvis nye oplysninger opstår.

Voksne informeres om indholdet af de gyserfilm de kan tilvælge at se under forsøget, så de har mulighed for at vælge dem fra på et kvalificeret grundlag

Børn, der deltager i forsøget, vil få mundtlig information og blive inddraget i samtalerne med forældrene om forsøget i det omfang det pågældende barn eller den unge kan forstå forsøgssituationen (Dette gælder dog ikke, hvis det vil skade barnet). Barnet eller den unges egne tilkendegivelser vil – i det omfang de er relevante – blive tillagt betydning.

Person der afgiver informationen til børnene, har kendskab til området og har pædagogiske forudsætninger for at afgive information til børn. Da der er tale om ikke-interventionsforsøg vil det være tilstrækkeligt med samtykke fra én indehaver af forældremyndigheden.

Hvis forsøgspersonen er 15-17 årige vil den unge også få skriftlig information om forsøget og give et selvstændigt samtykke til deltagelse i forsøget.

Samtalen tager udgangspunkt i den skriftlige deltagerinformation, og vil bl.a. indeholde oplysning om:

- Eventuelle forudsigelige risici, bivirkninger, komplikationer, ulemper, samt at der kan være uforudsigelige risici og belastninger knyttet til deltagelse i forsøget.

- Andre behandlingsmetoder (hvis forsøget også har behandlingsmæssigt sigte).

- At oplysninger fra om helbredsmæssige forhold, rent private forhold og andre fortrolige oplysninger kan videregives til og behandles af personer, som skal foretage en lovpligtig kvalitetskontrol af forsøget

- Forhold, som forsøgspersonen i øvrigt skønnes at være uvidende om, men som har betydning for forsøgspersonens stillingtagen, fx at vederlag til deltagerne er skattepligtige.

- Evt. andet relevant.

Efter informationssamtalen vil forsøgspersonen eller stedfortræder blive informeret,

- Hvis der under gennemførelsen af forsøget fremkommer nye oplysninger om effekt, risici, bivirkninger, komplikationer eller ulemper,

- Hvis forskningsprojektets forsøgsdesign ændres væsentligt i forhold til forsøgspersonens sikkerhed (gælder forsøgspersoner, der aktivt deltager i forsøget),

- Hvis der under gennemførelsen af forskningsprojektet fremkommer væsentlige oplysninger om forsøgspersonens helbredstilstand, medmindre forsøgspersonen utvetydigt har givet udtryk for, at den pågældende ikke ønsker dette,

- Om de resultater, der er opnået samt om eventuelle konsekvenser for den enkelte. Dette forudsætter, at det er praktisk muligt og forsøgspersonen ønsker dette.

- Hvis forsøget afbrydes, skal forsøgspersonen informeres om årsagen hertil

Hvis det ønskes kan forsøgspersonen blive informeret om de resultater projektet fremkommer med ved afslutning af forskningsprojektet. I øvrigt henvises der til National Videnskabsetisk Komités vejledning om rettigheder som forsøgsperson på: https://www.nvk.dk/forsoegsperson/dine-rettigheder-som-forsoegsperson.

Children

We are aware that certain precautions should be taken and followed in regards to enrolling children in a research study (MDR article 65). However, we are convinced that the study fulfills all the requirements listed in the MDR in this regard. It is of great importance to the project also to be able

to recruit children and young adults under the age of 18, because the heart rate features of children and adults are different. Also, epileptic seizures are very heterogeneous in their nature and some types of seizure are only seen in children. Therefore, it is crucial for this project to test if the seizure detection algorithms also are reliable for children. A seizure alarm system for children would be of great importance for both themselves and the parents. The importance of recruiting children to this specific project is furthermore specified and underlined in the attached documents from the Danish Epilepsy Association and pediatrician Professor, Dr.Med Christina Høi-Hansen from Institute of Clinical Medicine, Copenhagen University (16.01 & 16.02). Furthermore, the expected amount of children recruited in the study (50 children) will be sufficient to fulfill the requirements of a phase-3 study as it has been outlined in "Standards for testing and clinical validation of seizure detection devices"[17], as described above.

Sample size estimation

We expect to record 150 patients with more than 250 recorded seizures in total. See supplement 6 (04.08 sample size estimation) for further elaboration.

Timeframe of patient recruitment

The period of enrollment for each of the volunteering patients are only during the 1-5 days, when admitted to the video-EEG monitoring unit. The patient recruitment will be initiated 1/1 2022 (or as soon as the project is approved by the ethics committee) and end at 1/9 2024.

Data Collection

The recording and data collection will be done using the portable CE-marked ECG-device, C3+ Holter Monitor patch. The patients will be recorded during the whole 1-5 day period they are enrolled for video-EEG long term monitoring (LTM). The C3+ device will be placed on the patient as soon as possible after the video-EEG monitoring equipment has been placed and the written consent form has been signed. During the first day of the enrolment period patients will be invited to complete a standardized training session (exercise bike) to obtain the personalized threshold setting of the seizure detection algorithm, as HRV has been proved to change with physical exercise[19]. Likewise, the patients will also be invited to perform an algorithmic stress test and watch a horror movie (only adult patients) as cognitive stress is known to influence sudden changes in the HRV parameters[20–22]. If the patients are not capable of doing one or any of the tests or do not wish to do so, they are still invited to volunteer in the project, without doing the test(s). Children who the clinical doctors consider to be either too young or in any way unfit to do any of the tests will not be invited to do them. The tests will be used together with the first 24 hours ECG data to set the individual detection threshold

parameters for each of the patients. It will later be evaluated if the patients that did not do one or more of the tests will have a different outcome of seizure detection sensitivity or specificity.

The patients (and parents when the patient is a child) will be given specific instructions in how the seizure detection app works, how to use it and how to turn off the seizure alarm when the seizure detection threshold has been surpassed. So, when the patient is a child, it will be the parents who will be instructed in how to turn off the alarm. However, if the patient is a child <12 years old, the alarm will be muted during the entire stay, and if the patient is a child >12 years old or an adult the alarm will be muted during nighttime when sleeping which will prevent the patients from waking up due to the alarms. Also, the clinical staff monitoring the patients will be instructed in the process and how the seizure detection app works and to turn the alarm off if the patient are having a seizure with impaired consciousness and therefore not are able to do it by themselves. The clinical staff are already familiar with handling alarms during the video-EEG monitoring and an important part of the clinical examination is for the staff to test the patient during seizures to establish weather or not the patient has impaired awareness. The first 24 hours of ECG-recording data will be used to find the patients threshold of seizure detection as described in "The Seizure Detection Algorithm" in Investigators Brochure and in Jeppesen et.al 2019[6]. The seizure detection alarm app will be installed on smartphones Pixel 4a which have been purchased for the research project. The ECG-data and seizure loggings from the recordings will be saved both on the Cortrium C3+ device and on the smartphone for later posthoc analysis as well. This data will be transferred to a computer where the posthoc analysis will be done. The C3+ device will be detached the patient when the video-EEG monitoring equipment is also detached and the mobile phone will be delivered to the staff.

Analysis Strategy

Statistical analysis of the sensitivity and specificity of seizure detection by means of the HRV algorithm developed in (Jeppesen et al., Epilepsia 2019)[6] implemented on the smartphone device will be conducted for the whole enrollment period of 1-5 days. This will be done both individually and group-specifically (epilepsy form and type and age specific (children vs adults)).

Information from patient journals and treatment of personal information.

The clinical responsible doctors will use the patient journals do see if the patients enrolled for video-EEG monitoring are electable for inclusion of the study. Clinical specialist physicians will determine the time of seizure-onset and seizure type and focal grouping based on the video-EEG recordings. The clinical records will only be used by the clinical responsible in terms of the specific epilepsy

diagnose and nature of the epileptic focus. The clinical responsible will pass on this information to the principal investigator after the completion of the video-EEG recording.

The personal information of the participants will be protected as the law of "treatment of personal information and health law" (personoplysninger og sundhedsloven) prescribe it, and the study will be complied with the Data Protection Regulation and the Data Protection Act. The study is in compliance with the principles of good clinical practice in the field of clinical investigations of devices and the project will be registered in the Aarhus University data controller record. All data is collected to Aarhus University and no data will be distributed to any company.

Risk, side effects and inconvenience, short and long-term

The level of inconvenience and discomfort in participating in the study is considered to be highly limited, as the recordings in the project use the same time and space settings, as the video-EEG monitoring unit for which the patients have already been enrolled independently of our research study. A slight inconvenience or discomfort may occur for some patients when doing the tests (exercise, algorithmic stress test or watching a horror movie), however these tests are optional and the patients can at any time before or during the tests choose not to do any or all of them. Often these tests or other tests like these are done in the long-term video-EEG monitoring unit as a routine in order to either try to provoke seizures or interictal spikes, so the staff is used to handling all sorts of tests. Temporary skin irritation may occur at the location of the ECG-electrodes, however the exact same electrodes are used for recording for recording EMG and wired standard ECG during the video-EEG monitoring, so the inconvenience of this is considered highly limited. However, if any side effects should occur due to the project in any way this will be registered by the video-EEG monitoring staff, who are following the patients closely during the monitoring and the information will be passed on the principal investigator, who will report this in the annual safety report. If the patients have any form of abnormal ECG found from data from the ECG-patch we will follow the same procedures as when abnormal ECG is found on the mandatory wired ECG during the video-EEG monitoring.

Organization and Resources

Assistant Professor Jesper Jeppesen and Professor Sándor Beniczky, Dep of Clinical Medicine, Aarhus University (AU) & Dep. of Neurophysiology, Aarhus University Hospital (AUH) will prepare, conduct, evaluate results of and administer the project and preserve the cooperative agreements with all collaborators. Professor and MD Sándor Beniczky is both affiliated in Aarhus University and Danish Epilepsy Hospital creating the cooperation between the epilepsy monitoring

units in the project. Data processing, programming, signal processing, analysis and evaluation of the results will be carried out by cand.scient.med. PhD Jesper Jeppesen at the Dep. Neurophysiology, AUH and Dep. of Engineering (ENG), AU. Two or more M.Sc. students in biomedical engineering from ENG, AU will imbed the adaptive detection algorithm and create a user-friendly application which can be incorporated directly in smartphones.

A consultant and advisory board of senior researchers with expert knowledge in epilepsy, seizure detection research and signal processing has been established to optimize the recruiting, seizure detection algorithm and seizure alarm system. The advisory board consists of Prof. Emeritus DMSc Anders Fuglsang-Frederiksen, Dep of Clinical Medicine, AU; Ass. Prof. DMSc Jakob Christensen, Dep of Neurology, AUH and Ass. Prof. Peter Johansen, ENG, AU. The projects physiological measurements of the epileptic patients will be carried out at the Dep. of Clinical Neurology, AUH and the Dep. of Clinical Neurophysiology, Danish Epilepsy Center (DEC). The Dep. of Neurophysiology & Neurology, AUH, and the Dep. of Clinical Neurophysiology, DEC will contribute with EEG analysis and remuneration of the clinical staff involved in the project. Dep. of Engineering, AU will provide software, testing facilities and technical expertise.

The biomedical company, Cortrium will supply the portable ECG-device systems, C3+ Holter Monitor patch (at a favorable price), for online recording of the patients. An NDA has been made with Cortrium, where they have given access to the reading the ECG data online on the mobile platform.

Economy

The project has been initiated by Assistant Professor Jesper Jeppesen and Professor Sándor Beniczky from the Dep of Clinical Medicine, AU and Dep of Neurology, Aarhus University Hospital. The project have been fully founded by the Independent Research Fund (2.000.000,- kr.), which is administered by AU in a project account specified for the project. The fonds will be applied for salary of Assistant Professor Jesper Jeppesen, purchase of Cortrium C3+, mobile phones and all other expenses regarding the project. There is no association between the project leader and the Independent Research Fund. No financial fee will be given to the volunteers, neither of transportation nor remuneration of participation.

Dissemination and publication

The results will be published in international scientific peer-reviewed journals. Data will be pseudo anonymized and both negative as well as positive results will be published. The aim is to publish papers in Epilepsia, Seizure and IEEE Engineering in Medicine and Biology Society. Furthermore, the results and progress will be presented at scientific international conferences.

Ethics

The study fulfills the Helsinki II declaration and will be reported to The National Committee on Health Research Ethics in Denmark. ECG is a measurement that does not entail any risks for the patients involved. All patients are already enrolled for video/EEG examination, so no additional hospitalization is required by the project. The results of all the examinations regarding the epileptic disorder will be passed on to the project leader in order to find patterns of positive seizure detections with HRV and epilepsy form and type. It has been taking into consideration that the children and young adults under the age of 18 will be given the oral information in a pedagogic manner by the clinical responsible, who all have experience in working with children. Written information and consent forms are given to all patients and withdrawal from the project is possible at any time. In the written consent permission to use the patient's EEG, MEG and MR-scan data for analysis is also included. In the method section "Retningslinjer for afgivelse af mundtlig information" a detailed description of the procedure of recruitment, oral information and obtaining the consent is presented.

The level of inconvenience and discomfort in participating in the study is considered to be highly limited, as the recordings in the project use the same time and space settings, as the video-EEG monitoring unit for which the patients have already been enrolled independently of our research study. A slight inconvenience or discomfort may occur for some patients when doing the tests (exercise, algorithmic stress test or watching a horror movie), however these tests are optional and the patients can at any time before or during the tests choose not to do any or all of them.

If a portable seizure alarm system could be developed, it would be an important asset for patients with epilepsy[9]. A reliable alarm system would be high in demand worldwide and such could potentially be beneficial for many thousands of refractory epilepsy patients.

It is important for the project also to be able to recruit children and young adults under the age of 18, because the heart rate features of children and adults are different. Also, epileptic seizures are very heterogeneous in their nature and some types of seizure are only seen in children, therefore it is very important to verify that these seizure types can also be detected in this study. It is thus crucial for this project to test if the seizure detection algorithms also are reliable for detecting seizures in children. Scientifically, it is also genuine that the benefits which a seizure detection alarm will prove for the children is outwaiting the highly limiting inconvenience of participating in the study. In our previous study using a similar wearable ECG-device, there were no complaints from any of the participants (children or adults) and some of the patients even forgot they were wearing the device (see also Investigators Brochure for detailed description of previous studies). A seizure alarm system for children with epilepsy would of great importance for both themselves and the parents and caretakers of the children.

References

1 Christensen J, Vestergaard M, Pedersen MG, Pedersen CB, Olsen J, Sidenius P. Incidence and prevalence of epilepsy in Denmark. *Epilepsy Res* 2007; **76**: 60–5.

2 Shorvon S, Tomson T. Sudden unexpected death in epilepsy. *Lancet* 2011; **378**: 2028–38.

3 Hoppe C, Poepel A, Elger CE. Epilepsy: Accuracy of patient seizure counts. *Arch Neurol* 2007; **64**: 1595–9.

4 Jeppesen J, Beniczky S, Johansen P, Sidenius P, Fuglsang-Frederiksen A. Detection of epileptic seizures with a modified heart rate variability algorithm based on Lorenz plot. *Seizure* 2015; **24**: 1–7.

5 Jeppesen J, Beniczky S, Johansen P, Sidenius P, Fuglsang-Frederiksen A. Using Lorenz plot and Cardiac Sympathetic Index of heart rate variability for detecting seizures for patients with epilepsy. *Conf Proc IEEE Eng Med Biol Soc* 2014; **2014**: 4563–6.

6 Jeppesen J, Fuglsang-Frederiksen A, Johansen P, Christensen J, Wüstenhagen S, Tankisi H, *et al.* Seizure detection based on heart rate variability using a wearable electrocardiography device. *Epilepsia* 2019; **60**: 2105–13.

7 Jeppesen J, Fuglsang-Frederiksen A, Johansen P, Christensen J, Wüstenhagen S, Tankisi H, *et al.* Seizure detection using heart rate variability: A prospective validation study. *Epilepsia* 2020; : epi.16511.

8 Beniczky S, Jeppesen J. Non-electroencephalography-based seizure detection. *Curr Opin Neurol* 2019; **32**: 1.

9 Beniczky S, Wiebe S, Jeppesen J, Tatum WO, Brazdil M, Wang Y, *et al.* Automated seizure detection using wearable devices: A clinical practice guideline of the International League Against Epilepsy and the International Federation of Clinical Neurophysiology. *Clin Neurophysiol* 2021; **132**: 1173–84.

10 Mormann F, Andrzejak RG, Elger CE, Lehnertz K. Seizure prediction: the long and winding road. *Brain* 2007; **130**: 314–33.

11 Tomson T, Ericson M, Ihrman C, Lindblad LE. Heart rate variability in patients with epilepsy. *Epilepsy Res* 1998; **30**: 77–83.

12 Harnod T, Yang CCH, Hsin Y-L, Wang P-J, Shieh K-R, Kuo TBJ. Heart rate variability in patients with frontal lobe epilepsy. *Seizure* 2009; **18**: 21–5.

13 Harnod T, Yang CCH, Hsin Y-L, Shieh K-R, Wang P-J, Kuo TBJ. Heart rate variability in children with refractory generalized epilepsy. *Seizure* 2008; **17**: 297–301.

14 Evrengül H, Tanriverdi H, Dursunoglu D, Kaftan A, Kuru O, Unlu U, *et al.* Time and frequency domain analyses of heart rate variability in patients with epilepsy. *Epilepsy Res* 2005; **63**: 131–9.

15 Ansakorpi H, Korpelainen JT, Huikuri H V, Tolonen U, Myllylä V V, Isojärvi JIT. Heart rate dynamics in refractory and well controlled temporal lobe epilepsy. *J Neurol Neurosurg Psychiatry* 2002; **72**: 26–30.

16 Persson H, Kumlien E, Ericson M, Tomson T. Preoperative heart rate variability in relation to surgery outcome in refractory epilepsy. *Neurology* 2005; **65**: 1021–5.

17 Beniczky S, Ryvlin P. Standards for testing and clinical validation of seizure detection devices. *Epilepsia* 2018; **59**: 9–13.

18 Cortrium C3 Holter monitor. https://www.cortrium.com/.

19 Grossman P, Wilhelm FH, Spoerle M. Respiratory sinus arrhythmia, cardiac vagal control, and daily activity. *Am J Physiol Heart Circ Physiol* 2004; **287**: H728-34.

20 McDuff D, Gontarek S, Picard R. Remote Measurement of Cognitive Stress via Heart Rate Variability. 2014; : 3–6.

21 Castro MN, Vigo DE, Chu EM, Fahrer RD, de Achával D, Costanzo EY, *et al.* Heart rate variability response to mental arithmetic stress is abnormal in first-degree relatives of individuals with schizophrenia. *Schizophr Res* 2009; **109**: 134–40.

22 Kuehl LK, Deuter CE, Richter S, Schulz A, Rüddel H, Schächinger H. Two separable mechanisms are responsible for mental stress effects on high frequency heart rate variability : An intra-individual approach in a healthy and a diabetic sample. *Int J Psychophysiol* 2014. doi:10.1016/j.ijpsycho.2014.12.003.
